# Identifying diabetic ketoacidosis encounters at diagnosis among children with type 1 diabetes in claims data

**DOI:** 10.1210/jendso/bvag146

**Published:** 2026-06-30

**Authors:** R Brett McQueen, Nai-Chia Chen, Eric J Gutierrez, Kelly E Anderson, G Todd Alonso

**Affiliations:** University of Colorado Anschutz Medical Campus, Skaggs School of Pharmacy and Pharmaceutical Sciences, Aurora, CO 80045, USA; University of Colorado Anschutz Medical Campus, Skaggs School of Pharmacy and Pharmaceutical Sciences, Aurora, CO 80045, USA; University of Colorado Anschutz Medical Campus, Skaggs School of Pharmacy and Pharmaceutical Sciences, Aurora, CO 80045, USA; University of Colorado Anschutz Medical Campus, Skaggs School of Pharmacy and Pharmaceutical Sciences, Aurora, CO 80045, USA; University of Colorado, Anschutz Medical Campus, Barbara Davis Center for Diabetes, Aurora, CO 80403, USA

**Keywords:** diabetic ketoacidosis, real-world claims data, type 1 diabetes, healthcare resource utilization, burden

## Abstract

**Background:**

Quantify the performance of claims data in identifying diabetic ketoacidosis (DKA) cases and estimate healthcare resource utilization among newly diagnosed type 1 diabetes patients with and without DKA.

**Methods:**

We matched individuals <18 years of age from the Barbara Davis Center for Diabetes Registry to the Colorado all-payer claims database to estimate variation in the performance of claims (ie, type 1 diabetes and DKA codes in emergency department [ER] and inpatient [IP] settings) in identifying DKA cases from 2014 to 2019. We estimated the unadjusted and adjusted associations of laboratory-confirmed DKA with resource utilization using encounters and incidence rate ratios with 95% confidence intervals.

**Results:**

After applying algorithms for identifying type 1 diabetes with continuous enrollment criteria, n = 728 (62% match of the sample) represented an insured population residing in Colorado. Mean age at onset was approximately 9.48 years, with a proportion of DKA events at diagnosis of 56%. Strict coding definitions of DKA (DKA and type 1 diabetes) had lower sensitivity (63%) and higher specificity (76%) than looser definitions (type 1 diabetes or DKA), which had higher sensitivity (89%) and lower specificity (60%). Laboratory-confirmed DKA was associated with over 5 times the rate of IP encounters compared with no confirmed DKA (incidence rate ratio [IRR], 5.49; 95% confidence interval, 4.03-7.47) but fewer ER-only encounters (IRR, 0.36; 95% confidence interval, 0.24-0.54).

**Conclusion:**

Claims data may not capture all DKA encounters at type 1 diabetes diagnosis. Future research should provide sensitivity analyses across coding and settings to estimate the burden of DKA.

Diabetic ketoacidosis (DKA) at the onset of type 1 diabetes is common, dangerous, and expensive to treat. DKA events at diagnosis have increased up to an alarming 60% among all children diagnosed with type 1 diabetes in Colorado over the past decade [1-3]. Across the United States, the total number of discharges with a principal DKA diagnosis increased from 118 808 in 2003 to 188 965 in 2014, leading to an increase of approximately $3 billion in total spending to manage these patients [4]. Hospital admission charges per person can be up to $30,000, varying by length of stay, among other factors. Fortunately, mortality from DKA is rare; however, other complications may include cerebral and kidney injuries [5].

Studies in high-risk children have led to the consensus that presymptomatic type 1 diabetes in children should be identified early to allow more timely diagnosis of type 1 diabetes and avoidance of severe DKA events, resulting in better outcomes [6, 7]. Emerging evidence from multiple large-scale screening programs suggests screening, monitoring, and awareness initiatives are meeting a priori goals for reducing DKA at the onset of type 1 diabetes [8, 9]. While evidence on the clinical benefits of presymptomatic type 1 diabetes screening and education continues to emerge, key gaps remain for understanding the burden of DKA around the United States. The current and best available evidence around DKA rates at diagnosis of type 1 diabetes is limited to single tertiary care centers [1, 10, 11]. Given heterogeneity in demographics, geography, and socioeconomic status, DKA rates may vary widely across the United States. There is only 1 recent publication on costs attributable to DKA at diagnosis, and it is limited in scope by a lack of confirmation from clinical data from the EMR and specific to patients in private insurance plans only [12].

In Colorado, during the past 2 decades, the proportion of children presenting with DKA at initial diagnosis has increased from approximately 30% to 55% or 60% [1, 3, 13]. Evidence suggests the impact of DKA persists over longer time horizons, with a poorer long-term glycemic trajectory for those with DKA events at diagnosis as compared to those without DKA events at diagnosis [14-16]. Moreover, recent findings suggest DKA at diagnosis is associated with significantly higher use of health services [4, 12, 17]. However, the majority of existing literature focuses on the resources and expenditures during the event, either as a hospital stay or an emergency room (ER) visit, and not the resources used leading up to and after the event. Given the field can better predict the presymptomatic stages of type 1 diabetes, expanding this evidence base using claims data is crucial for future research and targeting interventions such as screening and monitoring [18].

All-payer claims databases (APCDs) are key data sources for understanding trends and drivers of clinical event rates and resource utilization outcomes in specific states within the United States [19]. However, there has been no national effort to estimate the quantitative range of bias in APCDs when identifying DKA rates at onset. Claims data were not designed for research purposes, but rather for insurance-related billing and reimbursement. This can cause challenges when identifying specific events in claims. For example, patients may move between health plans, so what may seem like a new onset DKA event within the claims data of a given health plan might be an existing patient with type 1 diabetes having a DKA event. Moreover, claims may miss moderate DKA events that do not require an ER visit or hospitalization but are still a significant clinical event for a patient. Validation with performance metrics is an important step to determine the degree of bias in claims data when identifying DKA events at type 1 diabetes onset. For example, claims-based diagnoses often do not include laboratory data and, without confirmation from electronic medical record (EMR) data, may not match the formal, clinical definition of DKA (pH < 7.3 or serum HCO_3_^−^ < 15 mmol/L) [20], with DKA occasionally overcalled in borderline cases and rarely missed by the initial provider. Moreover, a miscoded diagnosis of type 2 diabetes rather than type 1 may occur. Therefore, it is important to validate a subset of claims-based diagnoses by correlating them with clinical data. Our objective was to quantify the performance of the Colorado APCD in identifying DKA cases compared with laboratory-confirmed DKA, as well as estimating healthcare resource utilization among newly diagnosed type 1 diabetes patients with and without DKA.

## Methods

### Data source and study population

The EMR data at the Barbara Davis Center for Diabetes include type 1 diabetes patients with complete data for encounters occurring at the Barbara Davis Center and throughout the UCHealth system (ie, demographics, clinical characteristics, laboratory values, provider notes), representing over 90% of all Colorado children diagnosed with type 1 diabetes. This analysis builds on a 2020 publication by adding new patients, with the study period limited to the period of 2014 to 2019 to ensure a temporal match between EMR data from the Barbara Davis Center and the Colorado APCD [1]. As expected, using only EMR data, insurance status was poorly coded or unknown among 57% of patients. We linked the Colorado APCD to EMRs using medical record numbers and health plan numbers for each individual.

The Colorado APCD is the state's source of health care insurance claims, representing most covered lives across commercial health plans, Medicare, and Medicaid. The claims include important information about the encounter, including diagnosis, location, and services provided. The Colorado APCD is administered by the Center for Improving Value in Health Care [21]. Given that APCD data are only available for the subset of individuals seen at UCHealth centers (approximately 60% match), we matched only where patients had both EMRs plus claims data.

The complete inclusion criteria included type 1 diabetes diagnosis, sufficient medical records to rule in or out the presence of DKA at diagnosis [1], less than 18 years of age at diagnosis during a time window of 2014 to 2019 (ie, study time period from first data available in January 2014 up to December 2019), laboratory values for DKA including pH or HCO_3_, an insulin prescription (eg, therapeutic code 682008) within 6 months following diagnosis date, continuous enrollment for 6 months prediagnosis and 12 months postdiagnosis with no type 1 diabetes diagnosis prior to the index date. To be included in the analytic cohort, each individual required the full enrollment time period for baseline and follow-up within the calendar years of 2014 to 2019. For example, if an individual had an index date of February 1, 2014, they would not have the complete baseline time for inclusion in the analytic cohort. We relaxed continuous coverage criteria in a sensitivity analysis. Exclusions include gestational diabetes and adults with type 1 diabetes (≥18 years of age at diagnosis). These rules were developed to mirror similar claims-based analyses often used to estimate resource utilization or other outcomes important to characterize disease burden without the use of EMR records.

### Study design

**Figure 1:**
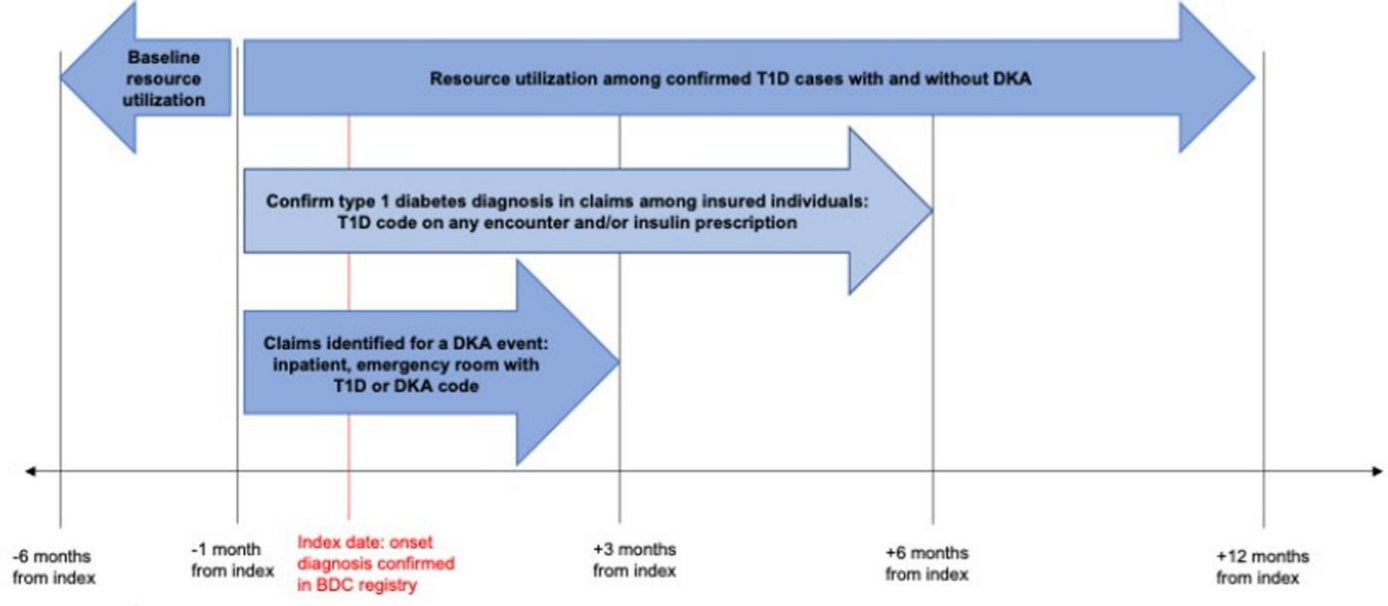
Claims for type 1 diabetes diagnoses and diabetic ketoacidosis events.

**Figure 2:**
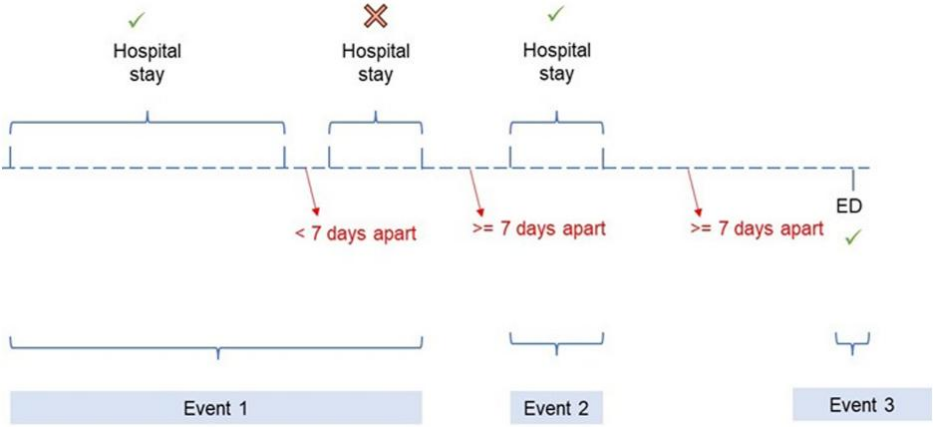
Distinct diabetic ketoacidosis encounters.

To capture DKA events in claims, we relied on the anchor of a provider-confirmed type 1 diabetes diagnosis available in the EMRs as the index date. As noted previously, each member of the primary cohort had continuous medical and pharmacy enrollment from the baseline of 6 months up to +12 months from the index date. We generated time windows that spanned 1 year prior to the index date and 1 year post-index date. We then estimated resource utilization across 3 separate time periods: baseline (−6 months to −1 month from index date), diagnosis window (−1 month to +3 months from index date), and follow-up (+3 months to +12 months from index date) (Fig. 1). As noted in the inclusion period, we confirmed a type 1 diabetes diagnosis through both a follow-up type 1 diabetes code attached to any claim and insulin utilization per existing algorithms. We then created unique encounters by ensuring each encounter was separated by at least 7 days so as not to double-count the same encounters (Fig. 2).

### Algorithm to identify DKA events in APCD

We relied on previously built algorithms comparing type 1 diabetes cases with and without the use of EMRs [22]. Specifically, Zhong et al found that using administrative claims (ie, type 1 diabetes diagnosis code and insulin prescription fill) to identify type 1 diabetes cases had sensitivity, specificity, and positive predictive value (PPV) of greater than 90%. We extended this analysis to include billing codes for DKA in different settings relevant to DKA management (ie, ER and inpatient [IP] encounters). Similar to other billing and payment data sources, the Colorado APCD provides claim-level data (ie, claims codes for services, pharmaceuticals, etc.) where patients may be repeated multiple times in 1 data set based on the myriad of services needed for treatment. Each code may be associated with a specific setting, diagnosis code, and provider, among other characteristics associated with an encounter. In this specific example, the algorithm identified 2 scenarios: (*a*) at least 1 type 1 diabetes code attached to an ER or IP visit; or (*b*) type 1 diabetes code and a DKA code attached to an ER or IP visit. Codes for type 1 diabetes identification include ICD-9-CM codes (for years 2014-2015) 250.01, 250.03, 250.11, 250.13, 250.21, 250.23, 250.31, 250.33, 250.41, 250.43, 250.51, 250.53, 250.61, 250.63, 250.71, 250.73, 250.81, 250.83, 250.91, and 250.93; ICD-10 codes E10.8, E10.9, E10.2, E10.3, E10.32, E10.33, E10.34, E10.25, E10.36, E10.37, E10.4, E10.5, E10.5, and E10.6; and ICD-10 codes specific to ketoacidosis or hyperglycemia: E10.10. E10.11, and E10.65.

### Algorithm validation against the gold standard

The primary measure of DKA used as the gold standard for comparison to claims-based DKA identification was based on laboratory data (using pH <7.3 or serum HCO_3_^−^<15 mmol/L) available in the Barbara Davis Center for Diabetes registry. We calculated sensitivity, specificity, PPV, and negative predictive value (NPV) to identify the performance of identifying DKA using claims data against the gold standard of laboratory data. Our goal was to balance high sensitivity and high specificity in identifying DKA events in claims data. The optimal algorithm was used to stratify groups and estimate excess resource utilization in the APCD from laboratory-confirmed DKA vs no DKA.

### Measures

Other variables in EMR records included basic demographics, including age at onset, sex, year of diagnosis, race/ethnicity, and insurance at diagnosis. APCD claims provided additional demographic data, including zip code, which we used to link to the Social Vulnerability Index (SVI) [23].

Resource utilization from APCD was specified by setting and type of resource use, including ER, IP, and OP encounters, as well as prescription medication utilization. We further split events by type 1 diabetes and all-cause to identify attributable events to type 1 diabetes. Among the all-cause events, we generated frequencies of all other codes recorded to identify symptoms associated with a type 1 diabetes IP or ER visit.

### Statistical analysis

Descriptive statistics were summarized for baseline characteristics, clinical characteristics, and resource utilization across laboratory-confirmed DKA at diagnosis (ie, DKA defined as pH <7.3 or serum HCO_3_^−^ < 15 mmol/L). To estimate differences in resource utilization across laboratory-confirmed DKA groups, we used Poisson regression, an approach useful when estimating the relationship between count outcomes and explanatory variables. The primary explanatory variable was laboratory-confirmed DKA to estimate the additional attributable resources used to manage and treat DKA. Other variables for adjustment included sex, insurance status, social vulnerability, and year of diagnosis. The adjusted analyses report incidence rate ratios (IRRs) with 95% confidence intervals. An IRR of 1 indicates similar resource use among both groups, whereas an IRR greater than 1 suggests an increased risk of resource utilization associated with a DKA event. Data management (and analytics) was performed using SAS version 9.4 and Stata version 15.1.

### Sensitivity analyses

Our primary study population included continuously enrolled individuals because of existing algorithms in claims that reduce the risk of identifying existing type 1 diabetes patients who had previously been diagnosed in other states or insurance providers. This primary cohort also represents one that can be tracked and monitored in potential screening and monitoring programs after identification of the risk of type 1 diabetes using islet autoantibody or genetic risk score screening. However, these algorithms may miss individuals diagnosed outside of their provider networks (eg, children on vacation with their families). Therefore, in addition to providing performance metrics for the subgroup of continuously enrolled individuals, we provide estimates for the population not continuously enrolled.

## Results

**Figure 3:**
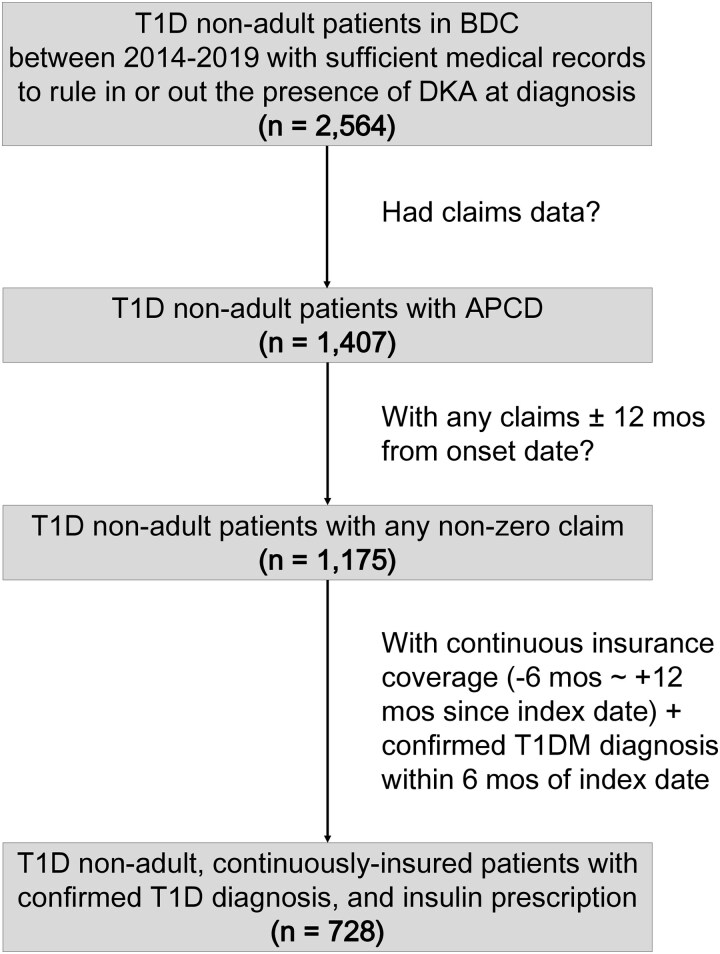
Flow chart of patients for analysis. A sensitivity analysis was run on the population of 1175 to represent a snapshot cohort without continuous enrollment.

There were 2564 nonadult patients in the Barbara Davis Center for Diabetes Registry during the time period of 2014 to 2019 with sufficient medical records to rule in or out the presence of DKA at diagnosis. Of those, 1407 were matched in the Colorado APCD (Fig. 3). A total of 1175 had some type of claim within 12 months of the diagnosis date recorded in the Barbara Davis Center for Diabetes Registry. Among the 1175 patients with any claim, 728 patients had continuous enrollment with a future type 1 diabetes diagnosis and insulin use confirmed through claims. We reviewed provider notes to identify whether patients were expected to have claims for the 232 who did not have any claims, even with plan numbers in Colorado APCD. We found 25 with no insurance on file and 13 with parents residing in different states with varying insurance. The remaining 194 were expected to have insurance claims, but did not have any claims in the Colorado APCD.

Demographic and clinical characteristics were similar across laboratory-confirmed DKA and no-DKA status (Table 1). There were no differences between laboratory-confirmed DKA groups by sex with an approximately even estimate of females and males (*P* = .17), race/ethnicity with majority non-Hispanic white at 60% (*P* = .16), and year of diagnosis, which was consistent across groups (*P* = .84). Most patients were in Medicaid (63% DKA vs 53% no DKA) and private plans (33% for DKA vs 43% no DKA), but this difference was not significant. The biggest difference between laboratory-confirmed DKA and no DKA was the linked SVI with an even distribution across SVI in the no-DKA group, but a much more uneven distribution in the DKA group (*P* = .008). For example, of the total group with laboratory-confirmed DKA, 17% were in quartile 1 (lowest vulnerability) as compared with 30% in quartile 4 (highest vulnerability). The no-DKA group had an even distribution of approximately 25% across quartiles.

**Table 1:** Baseline demographics of continuous enrollment cohort

Characteristic	No DKA (n = 320)	Laboratory-confirmed DKA (n = 408)	Total (n = 728)	*P*-value
Sex, n (%)				.171
Female	146 (45.6)	207 (50.7)	353 (48.5)	
Male	174 (54.4)	201 (49.3)	375 (51.5)	
Race/ethnicity, n (%)				.158
American Indian/Alaska Native	0 (0.0)	3 (0.7)	3 (0.4)	
Asian	2 (0.6)	1 (0.2)	3 (0.4)	
Hispanic	60 (18.8)	99 (24.3)	159 (21.8)	
More than 1 race	6 (1.9)	10 (2.5)	16 (2.2)	
Native Hawaiian/Other	0 (0.0)	1 (0.2)	1 (0.1)	
Non-Hispanic Black	22 (6.9)	34 (8.3)	56 (7.7)	
Non-Hispanic White	211 (65.9)	227 (55.6)	438 (60.2)	
Other	7 (2.2)	9 (2.2)	16 (2.2)	
Unknown	12 (3.8)	24 (5.9)	36 (5.0)	
Year of diagnosis, n (%)				.843
2014	49 (15.3)	65 (15.9)	114 (15.7)	
2015	48 (15.0)	68 (16.7)	116 (15.9)	
2016	61 (19.1)	72 (17.6)	133 (18.3)	
2017	51 (15.9)	73 (17.9)	124 (17.0)	
2018	54 (16.9)	70 (17.2)	124 (17.0)	
2019	57 (17.8)	60 (14.7)	117 (16.1)	
Insurance category at diagnosis, n (%)				.081
Medicaid	168 (52.5)	257 (63.0)	425 (58.4)	
Military plans	2 (0.6)	2 (0.5)	4 (0.6)	
Unknown	9 (2.8)	10 (2.5)	19 (2.6)	
Private	136 (42.5)	133 (32.6)	269 (37.0)	
Not yet insured at diagnosis	5 (1.6)	6 (1.5)	11 (1.5)	
Age at onset, mean (SD)	9.80 (4.34)	9.23 (4.35)	9.48 (4.35)	.078
Social Vulnerability Index, n (%)				.008
1 (lowest vulnerability)	78 (24.9)	65 (16.5)	143 (20.2)	
2	81 (25.9)	87 (22.1)	168 (23.8)	
3	72 (23.0)	111 (28.2)	183 (25.9)	
4 (highest vulnerability)	82 (26.2)	131 (33.3)	213 (30.1)	

Abbreviations: DKA, diabetic ketoacidosis.

We calculated performance for two claims-based algorithms against the gold standard registry definition with laboratory data: (*a*) at least 1 type 1 diabetes code attached to an ER or IP visit or (*b*) type 1 diabetes code and a DKA code attached to an ER or IP visit. The performance of algorithm 1 had 89% sensitivity, 60% specificity, 74% PPV, and 81% NPV. The performance of algorithm 2 had 63% sensitivity, 76% specificity, 79% PPV, and 59% NPV. Algorithm 1 identified 364 true-positive DKA events with 128 false positives, whereas algorithm 2 identified 258 true positive DKA events with 69 false-positive events.

There were no differences in baseline resource utilization for ER visits or IP encounters across laboratory-confirmed DKA (Table 2) defined through the time window of –6 months to –1 month prediagnosis date. There were 9 individuals with at least 1 type 1 diabetes- or DKA-related IP encounter at baseline, suggesting the diagnosis in the Barbara Davis Center registry came after an IP encounter (see the following sensitivity analysis). Significant differences in resource utilization at baseline were specific to OP encounters and prescription medication utilization. The no-DKA group had a mean number of OP encounters of 3.76 compared to the DKA group of 2.61 (*P* = .001). The no DKA group had a mean unique prescription medication count with refills of 1.41 compared to 0.91 in the DKA group (*P* = .0002).

**Table 2:** Baseline healthcare resource encounters (6 months to –1-month prediagnosis)

Resource utilization	No DKA (n = 320)	Laboratory-confirmed DKA (n = 408)	Total (n = 728)	*P*-value*^a^*
Type 1 diabetes- or DKA-related inpatient visits, n (%)				.585
0 Visits	315 (98.4)	404 (99.0)	719 (98.8)	
1 Visit	3 (0.9)	2 (0.5)	5 (0.7)	
2+ Visits	2 (0.6)	2 (0.5)	3 (0.4)	
Length of stay, mean (SD) (days)	0.08 (0.99)	0.13 (1.64)	0.11 (1.39)	.679
Type 1 diabetes- or DKA-related ER visits alone, n (% yes)	0 (0.0%)	1 (0.3%)	1 (0.1%)	—
Type 1 diabetes- or DKA-related outpatient visits, mean (SD)	0.35 (2.04)	0.41 (2.73)	0.39 (2.45)	.739
All-cause inpatient visits, n (%)				.438
0 visits	309 (96.6)	400 (98.0)	709 (97.4)	
1 visit	9 (2.8)	7 (1.7)	16 (2.2)	
2+ visits	2 (0.6)	1 (0.2)	3 (0.4)	
All-cause LOS, mean (SD) (days)	0.15 (1.00)	0.29 (3.71)	0.23 (2.85)	.512
All-cause ER visits (alone), n (%):				.712
0 visits	233 (72.8)	301 (73.8)	534 (73.4)	
1 visit	60 (18.8)	79 (19.4)	139 (19.1)	
2+ visits	27 (8.4)	28 (6.8)	55 (7.6)	
All-cause outpatient visits, mean (SD)	3.76 (5.55)	2.61 (4.01)	3.11 (4.78)	.001
Unique prescription medications with refills—total, mean (SD)	0.75 (1.74)	0.40 (0.99)	0.55 (1.38)	.001
Unique prescription medications with no refills, mean (SD)	1.41 (2.01)	0.91 (1.61)	1.13 (1.81)	.0002

Percentages are calculated within DKA status groups.

Abbreviations: DKA, diabetic ketoacidosis; ER, emergency room; IP, inpatient; LOS, length of stay (days).

^*a*^ *P*-values from Pearson χ^2^ tests for categorical variables and *t*-tests for continuous variables.

**Figure 4:**
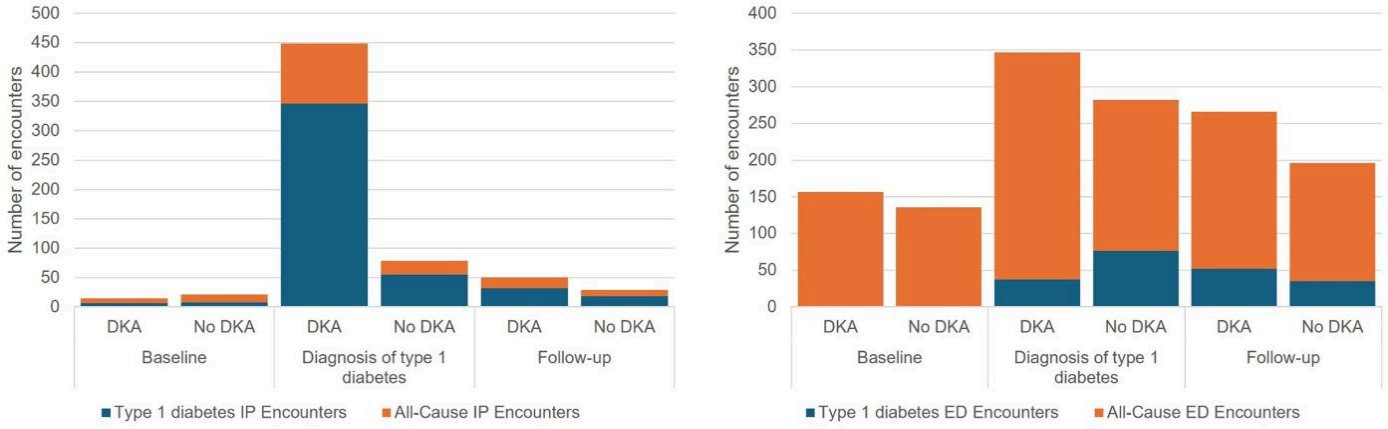
Inpatient and emergency department encounters by diabetic ketoacidosis status and time period. Baseline period is from –6 months to –1 month from index date (ie, diagnosis date confirmed in Barbara Davis Center Registry); index date is –1 month from index date to +3 months from index date; and follow-up is from +3 months from index date to +12 months from index date.

There were significant differences during the diagnosis window (−1 month to +3 months from diagnosis) for type 1 diabetes-related IP and ER encounters (Table 3 and Fig. 4) among the laboratory-confirmed DKA group compared to the no DKA group. The laboratory-confirmed DKA group was treated primarily in IP settings, with 340 (83%) individuals with at least 1 IP encounter with a type 1 diabetes code compared with 55 (17%) individuals in the no-DKA group (*P* < .001). Of those with a type 1 diabetes-related IP encounter, length of stay was significantly higher in the laboratory-confirmed DKA group with an average of over 5 days compared with approximately 1 day in the no-DKA group (*P* < .001). When expanding beyond type 1 diabetes-related encounters, 101 (25%) individuals had at least 1 all-cause IP encounter in the DKA group compared to 19 (6%) in the no DKA group (*P* < .001). All-cause ER encounters were similar between the 2 groups, with over 40% of both groups experiencing an all-cause ER encounter (*P* = .06).

**Table 3:** Healthcare resource encounters near diagnosis date (1 month prior to and 3 months post confirmed diagnosis date)

Resource Utilization	No DKA (n = 320)	Laboratory-confirmed DKA (n = 408)	Total (n = 728)	*P*-value*^a^*
Type 1 diabetes-related inpatient visits, n (%)				<.001
0 Visits	265 (82.8)	68 (16.7)	333 (45.7)	
1 Visit	55 (17.2)	334 (81.9)	389 (53.4)	
2+ Visits	0 (0.0)	6 (1.5)	6 (0.8)	
Length of stay, mean (SD), days	0.91 (4.34)	5.74 (15.3)	3.62 (12.1)	<.001
Type 1 diabetes-related ER visits alone, n (%)				<.001
0 Visits	245 (76.6)	374 (91.7)	619 (85.1)	
1 Visit	74 (23.1)	31 (7.6)	105 (14.4)	
2 Visits	1 (0.31)	3 (0.74)	4 (0.55)	
Type 1 diabetes-related outpatient visits, mean (SD)	4.38 (2.11)	4.61 (2.15)	4.51 (2.13)	.165
All-cause inpatient visits, n (%):				<.001
0 Visits	301 (94.1)	307 (75.3)	608 (83.5)	
1 Visit	16 (5.0)	100 (24.5)	116 (15.9)	
2+ Visits	3 (0.9)	1 (0.2)	4 (0.6)	
All-cause LOS, mean (SD), days	0.31 (1.72)	1.73 (10.7)	1.10 (8.11)	.019
All-cause ER visits (alone), n (%)				.062
0 Visits	188 (58.8)	201 (49.3)	389 (53.4)	
1 Visit	99 (30.9)	157 (38.5)	256 (35.2)	
2+ Visits	33 (10.3)	50 (12.3)	83 (11.4)	
All-cause outpatient visits, mean (SD)	2.54 (2.69)	2.30 (2.30)	2.41 (2.48)	.191
Unique prescription medications with refills—total, mean (SD)	2.70 (2.20)	2.73 (2.32)	2.72 (2.27)	.843
Unique prescription medications with no refills, mean (SD)	3.78 (2.48)	3.32 (2.31)	3.52 (2.40)	.011

Percentages are calculated within DKA status groups.

Abbreviations: DKA, diabetic ketoacidosis; ER, emergency room; IP, inpatient; LOS, length of stay (days).

^*a*^ *P*-values from Pearson chi-square tests for categorical variables and *t*-tests for continuous variables.

Differences across time between laboratory-confirmed DKA groups were most pronounced for IP encounters during the diagnosis window (Fig. 4), whereas ER visits were higher among both groups across the baseline, diagnosis window, and follow-up time periods. Additionally, there were many ER encounters without a type 1 diabetes or DKA code. We analyzed other codes attached to all-cause ER encounters to identify any codes related to type 1 diabetes. Of all the codes used on all-cause ER visits, we found 11 codes most frequently used (>1%), with a total of approximately 20% of all codes recorded related to diabetes or symptoms that may manifest as related to diabetes: E119 (T2D without complications), Z0012 (Encounter for routine child health examination), Z23 (Encounter for immunization), F840 (Autistic disorder), 25 000 (Incision on forearm), E139 (Other specified diabetes mellitus), V202 (Unspecified motorcycle rider injured in collision with pedestrian or animal nontraffic accident), J029 (Acute pharyngitis unspecified), R6889 (General symptoms and signs), and E1165 (T2D with hyperglycemia).

After adjusting for sex, insurance status, social vulnerability, and year of diagnosis, the primary explanatory variable of laboratory-confirmed DKA was significantly associated with increased IP encounters (Table 4) and fewer ER encounters. Specifically, laboratory-confirmed DKA was associated with over 5× the rate in the DKA group compared to the no DKA group (IRR, 5.49; 95% confidence interval, 4.03-7.47). Laboratory-confirmed DKA was also associated with fewer ER encounters (IRR, 0.36; 95% confidence interval, 0.24-0.54). No other explanatory variables reached statistical significance.

**Table 4:** Adjusted type 1 diabetes-specific and all-cause health care resource utilization

Variable	Type 1 diabetes or DKA code (laboratory-confirmed DKA vs no DKA)		All-cause (laboratory-confirmed DKA vs no DKA)	
	IRR (95% CI)	*P*-value	IRR (95% CI)	*P*-value
Inpatient encounters	5.49 (4.03-7.47)	<0.001	3.28 (2.03-5.29)	<.001
Emergency room encounters	0.36 (0.24-0.54)	<0.001	1.11 (0.91-1.36)	.273
Outpatient encounters	1.01 (0.94-1.08)	0.774	0.84 (0.77-0.93)	.001
Prescription medications with refills	N/A*^a^*	N/A	1.02 (0.94-1.12)	.576

Incidence rate ratio estimates were calculated after controlling for sex, insurance status, social vulnerability, and year of diagnosis.

Abbreviations: 95% CI, 95% confidence interval; DKA, diabetic ketoacidosis; N/A, not applicable.

^*a*^All prescription medications, including those not specific to type 1 diabetes.

**Figure 5:**
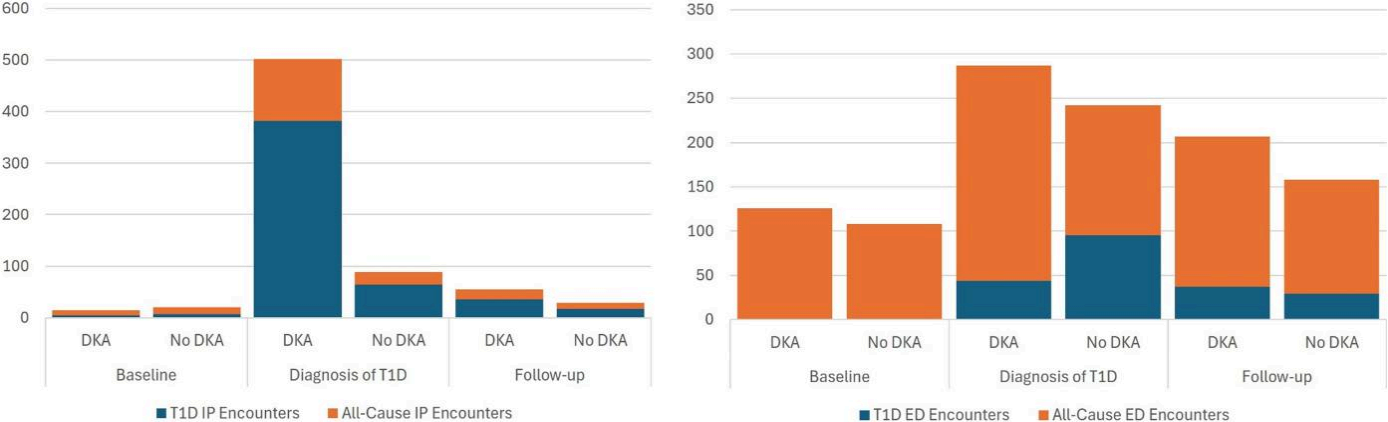
Inpatient and emergency department encounters by diabetic ketoacidosis status and time period for the noncontinuously enrolled cohort. Baseline period is from –6 months to –1 month from index date (ie, diagnosis date confirmed in Barbara Davis Center Registry); index date is –1 month from index date to +3 months from index date; and follow-up is from +3 months from index date to +12 months from index date.

We ran a sensitivity analysis using the cohort of 1175 individuals without continuous enrollment. Table 5 provides baseline estimates, which were mostly consistent with the continuous cohort, but with the exception of significant differences between race and ethnicity, as well as insurance status at diagnosis, in addition to SVI across DKA groups. The performance of algorithm 1 (type 1 diabetes or DKA code) had a sensitivity 77%, specificity 75%, PPV 73%, and NPV 80%. The performance of algorithm 2 (type 1 diabetes and DKA code) had a sensitivity 54%, specificity 88%, PPV 79%, and NPV 69%. Figure 5 describes the unadjusted IP and ER encounters. Expanding to the noncontinuous cohort led to an additional 36 type 1 diabetes-related IP encounters that were confirmed as true-positive DKA cases and 64 IP encounters overall across both DKA and no DKA groups during the diagnosis window. Table 6 summarizes the adjusted analysis, which is consistent with the findings in the continuous cohort. Laboratory-confirmed DKA was associated with increased type 1 diabetes-related (IRR, 7.49; 95% confidence interval, 5.62-9.97) and all-cause IP encounters (IRR, 4.49; 95% confidence interval, 2.90-6.95). Laboratory-confirmed DKA was associated with fewer type 1 diabetes-related ER encounters (IRR, 0.50; 95% confidence interval, 0.34-0.73) but increased all-cause ER encounters (IRR, 1.43; 95% confidence interval, 1.19-1.73).

**Table 5:** Demographics of noncontinuously enrolled cohort

Characteristic	No DKA (n = 636)	DKA (n = 539)	Total (n = 1175)	*P*-value
Sex, n (%)				.293
Female	311 (48.9)	247 (45.8)	558 (47.5)	
Male	325 (51.1)	292 (54.2)	617 (52.5)	
Race/Ethnicity, n (%)				<.001
American Indian/Alaska Native	0 (0.0)	4 (0.7)	4 (0.3)	
Asian	7 (1.1)	1 (0.2)	8 (0.7)	
Hispanic	88 (13.8)	121 (22.4)	209 (17.8)	
More than one Race	15 (2.4)	11 (2.0)	26 (2.2)	
Native Hawaiian/Other	2 (0.3)	1 (0.2)	3 (0.3)	
Non-Hispanic Black	36 (5.7)	39 (7.2)	75 (6.4)	
Non-Hispanic White	441 (69.3)	315 (58.4)	756 (64.3)	
Other	19 (3.0)	11 (2.0)	30 (2.6)	
Unknown	28 (4.4)	36 (6.7)	64 (5.4)	
Year of diagnosis, n (%)				.970
2014	103 (16.2)	86 (15.9)	189 (16.1)	
2015	104 (16.4)	95 (17.6)	199 (16.9)	
2016	120 (18.9)	96 (17.8)	216 (18.4)	
2017	103 (16.2)	92 (17.1)	195 (16.6)	
2018	110 (17.3)	95 (17.6)	205 (17.5)	
2019	96 (15.1)	75 (13.9)	171 (14.6)	
Insurance category at diagnosis, n (%)				<.001
Medicaid	208 (32.7)	288 (53.4)	496 (42.2)	
Military plans	9 (1.4)	4 (0.7)	13 (1.1)	
Unknown	31 (4.9)	22 (4.1)	53 (4.5)	
Private	382 (60.1)	213 (39.5)	595 (50.6)	
Not yet insured at diagnosis	6 (0.9)	12 (2.2)	18 (1.5)	
Age at onset, mean (SD)	9.69 (4.21)	9.32 (4.38)	9.52 (4.29)	.141
Social Vulnerability Index, n (%)				<.001
1 (lowest vulnerability)	187 (30.7)	95 (18.5%)	282 (25.1%)	
2	152 (25.0)	127 (24.8)	279 (24.9)	
3	139 (22.8)	141 (27.5)	280 (25.0)	
4 (highest vulnerability)	131 (21.5)	150 (29.2)	281 (25.0)	

Abbreviations: DKA, diabetic ketoacidosis.

**Table 6:** Adjusted T1D-specific and all-cause health care resource utilization for noncontinuously enrolled cohort

Variable	Type 1 diabetes or DKA code (laboratory-confirmed DKA vs no DKA)		All-cause (laboratory-confirmed DKA vs no DKA)	
	IRR (95% CI)	*P*-value	IRR (95% CI)	*P*-value
Inpatient encounters	7.49 (5.62-9.97)	<.001	4.49 (2.90-6.95)	<.001
Emergency room encounters	0.50 (0.34-0.73)	<.001	1.43 (1.19-1.73)	<.001
Outpatient encounters	1.32 (1.24-1.12)	<.001	1.08 (0.98-1.18)	.097
Prescription medications with refills	N/A*^a^*	N/A	1.29 (1.19-1.41)	<.001

IRR estimates were calculated after controlling for sex, insurance status, social vulnerability, and year of diagnosis.

Abbreviations: 95% CI, 95% confidence interval; DKA, diabetic ketoacidosis; IRR, incidence rate ratio; N/A, not applicable.

^*a*^All prescription medications, including those not specific to T1D.

## Discussion

We quantified the performance of the APCD in identifying DKA cases and estimating health care resource utilization among youth with newly diagnosed type 1 diabetes with 2 algorithms. Using a less strict algorithm to identify DKA (algorithm 1, at least 1 type 1 diabetes code attached to an ER or IP visit), we found high sensitivity (89%) but low specificity (60%), meaning that claims data identified 40% of no-DKA cases as having DKA. With the stricter approach (type 1 diabetes code and a DKA code attached to an ER or IP visit), sensitivity for identifying DKA dropped to 63% while specificity rose to 76%. Together, the performance of these 2 algorithms demonstrates the challenges in using claims data to identify DKA accurately.

Our findings on health care resource utilization are consistent with previous research using claims data or IP samples for DKA at diagnosis. For example, Saydah et al found individuals with commercial insurance who had DKA at diagnosis used fewer health services before diagnosis and higher medical costs surrounding and after the diagnosis time period [12]. Our findings were similar in that OP visits and prescription medication utilization were lower at baseline before diagnosis for those in the DKA group compared to those in the no DKA group at diagnosis. Additionally, we found similar results that DKA was higher among individuals insured through Medicaid as compared to private insurance [4]. Our analysis advances this work through an assessment of all insured individuals with a link to EMR data to assess the performance of claims.

Our findings have significant implications for establishing the economic burden of DKA for investment in interventions designed to reduce DKA, such as presymptomatic screening and monitoring. Previous evidence suggests baseline DKA rates and resource use are determining factors for estimating the value of screening programs [8]. In particular, programs may use APCDs to target local screening efforts for 2 main purposes: (*a*) investing in screening programs in high resource use areas where DKA is a major public health problem and (*b*) establishing a baseline for estimating potential future treatment effects of population-wide screening programs. However, our findings demonstrate that decision makers should be cautious in relying on APCDs and any source of billing and payment data to inform screening efforts.

For example, strict definitions around coding and continuous enrollment can decrease sample size and impact population-level estimates of the burden of DKA at diagnosis. For example, we initially matched 1407 patients between EMR data and claims data; however, only 1175 had any claims in the Colorado APCD. Further, among those 1175, only 728 were continuously enrolled. This large drop in sample size is not surprising, as recent evidence found a wide range of 30% to 66% of children had gaps in coverage (ie, were uninsured) after starting on Medicaid; only 26% were continuously insured throughout their entire childhood without starting on or switching to Medicaid [24]. Our findings are consistent with this recent evidence and led to missing 36 type 1 diabetes-related IP encounters that were confirmed as true positive DKA cases and 64 IP encounters overall across both DKA and no DKA groups during the diagnosis window. There are tradeoffs to expanding definitions, as screening programs largely hinge on being able to monitor children and adolescents over time to identify symptoms early before DKA. In the noncontinuous cohort, we found additional follow-up resource utilization that otherwise would have been missed with strict continuous enrollment criteria. Depending on the objectives, we recommend presenting sensitivity analyses around noncontinuously enrolled cohorts.

As expected, strict coding around DKA claims had low sensitivity and high specificity as compared to broader coding of any type 1 diabetes or DKA claim, which had high sensitivity and low specificity. Both algorithms can be useful for estimating the burden of DKA at diagnosis and any potential treatment effects from screening and monitoring programs. Additionally, variation in coding by setting produced mixed results associated with DKA at diagnosis. While ER encounters were similar among DKA and no DKA groups, it was clear that many of the symptoms associated with the initial presentation of type 1 diabetes were treated based on a mix of codes associated with type 2 diabetes and general encounters (eg, missed diagnoses). These findings suggest future researchers should provide multiple sensitivity analyses based on both algorithm coding and setting, presenting all-cause as well as type 1 diabetes-specific findings in order to estimate the potential scale of resource use for DKA encounters.

While we quantified health burden directly attributable to type 1 diabetes by using integrated EMR-linked claims data, there are gaps in the evidence base on cost drivers. Our linkage allowed us to anchor the analysis in laboratory-confirmed DKA cases (ie, gold standard), avoiding the common “overcalling” found in US administrative data, where suspected conditions are often coded as confirmed [25]. However, this economic footprint is influenced by factors our data cannot fully capture, including socioeconomic barriers beyond what's covered in the SVI. These findings suggest that reducing the DKA burden requires nuanced policy and clinical approaches that address both the diagnosis-related risks and the systemic barriers that lead to healthcare utilization. By analyzing both continuously and noncontinuously insured populations, this study informs the tradeoffs between high-sensitivity surveillance in well-tracked groups vs prioritizing high-specificity screening in under-resourced areas where social vulnerability may be most prevalent.

There are limitations to this analysis that should be noted. We did not match between claims and EMRs at 100%, in part due to linking EMR data with claims. This limitation is important to consider from a generalizability perspective. First, losing a large proportion of patients outside the UCHealth system inevitably reduces the generalizability to the population seen at academic medical centers and affiliates. Second, choosing a continuously insured population mitigates differential bias across groups (eg, 96% in both groups had type 1 diabetes claims up to 12 months from onset), but it leaves a substantial part of the population out of the analysis. This is in part why we chose to conduct a sensitivity analysis without the continuously insured population. However, caution should be taken when interpreting the sensitivity analysis despite similarities in the findings, as differential rates of utilization were observed between both groups during the 12-month follow-up period (eg, 89% had any type 1 diabetes claim in the laboratory-confirmed DKA group but only 64% in the no-DKA group). The differences are largely driven by insurance coverage, with a substantial part of the no DKA group without insurance after onset. Therefore, results from the sensitivity analysis are most important to interpret within the onset time window where patients arrive for urgent treatment for a DKA event and eventually leave the system (eg, in Colorado for vacation, moved out of the state, etc.). Further, children without insurance in the months prior to or following diagnosis were not included, nor were other children without insurance throughout the study time period.

Future research should focus on the cost implications for children diagnosed with type 1 diabetes who remain uninsured, especially in the current climate where Medicaid funding cuts and increased administrative burden to justify Medicaid eligibility will make it harder to obtain and maintain public insurance coverage. While we included SVI as a measure of socioeconomic status, it does depend on a link to zip codes. For example, large rural areas may produce the same SVI quartile, whereas urban areas are very heterogeneous in socioeconomic status. Finally, this study is not necessarily generalizable in terms of the main resource utilization findings (ie, the association of DKA with resource utilization). However, billing and payment data are often consistent across the United States, especially across APCDs in other states.

APCDs and, more broadly, claims data can be useful for estimating resource utilization for type 1 diabetes diagnoses with and without DKA despite variation in the performance of existing algorithms. However, claims data may not capture all DKA encounters at diagnosis. Future researchers should provide public health decision makers with a wide range of sensitivity analyses to inform decisions around the expansion of interventions designed to reduce DKA at diagnosis.

## Data Availability

Datasets generated during and/or analyzed during the current study are not publicly available.

## References

[1] Alonso GT, Coakley A, Pyle L, Manseau K, Thomas S, Rewers A. Diabetic ketoacidosis at diagnosis of type 1 diabetes in Colorado children, 2010–2017. Diabetes Care. 2019;43(1):117‐121. 31601639 10.2337/dc19-0428 PMC6925579

[2] Alonso GT, Murphy C, Pyle L, Thomas S, Ohman-Hanson R, Rewers A. Increased prevalence of diabetic ketoacidosis among Colorado children at diagnosis of type 1 diabetes during the COVID-19 pandemic lockdown resolves after reopening. Diabetes Technol Ther. 2021;23(9):663‐664. 33835859 10.1089/dia.2021.0062

[3] Alonso GT, Reinauer C, Williams GM, et al Regional deprivation and diabetic ketoacidosis at type 1 diabetes diagnosis in children and adolescents: international comparison among 6 countries. Horm Res Paediatr. 2026;99:591‐598. 39667350 10.1159/000543139 PMC12159260

[4] Desai D, Mehta D, Mathias P, Menon G, Schubart UK. Health care utilization and burden of diabetic ketoacidosis in the U.S. over the past decade: a nationwide analysis. Diabetes Care. 2018;41(8):1631‐1638. 29773640 10.2337/dc17-1379

[5] Glaser N, Fritsch M, Priyambada L, et al ISPAD clinical practice consensus guidelines 2022: diabetic ketoacidosis and hyperglycemic hyperosmolar state. Pediatr Diabetes. 2022;23(7):835‐856. 36250645 10.1111/pedi.13406

[6] American Diabetes Association Professional Practice Committee . 2. Diagnosis and classification of diabetes: standards of care in diabetes-2025. Diabetes Care. 2025;48(1 Suppl 1):S27‐s49. 39651986 10.2337/dc25-S002 PMC11635041

[7] Sooy M, Pyle L, Alonso GT, et al Lower prevalence of diabetic ketoacidosis at diagnosis in research participants monitored for hyperglycemia. J Clin Endocrinol Metab. 2024;110(1):e80‐e86. 38470864 10.1210/clinem/dgae158 PMC11651691

[8] McQueen RB, Geno Rasmussen C, Waugh K, et al Cost and cost-effectiveness of large-scale screening for type 1 diabetes in Colorado. Diabetes Care. 2020;43(7):1496‐1503. 32327420 10.2337/dc19-2003 PMC7305000

[9] Ziegler AG, Kick K, Bonifacio E, et al Yield of a public health screening of children for islet autoantibodies in Bavaria, Germany. JAMA. 2020;323(4):339‐351. 31990315 10.1001/jama.2019.21565 PMC6990943

[10] Wolf RM, Noor N, Izquierdo R, et al Increase in newly diagnosed type 1 diabetes in youth during the COVID-19 pandemic in the United States: a multi-center analysis. Pediatr Diabetes. 2022;23(4):433‐438. 35218124 10.1111/pedi.13328 PMC9115477

[11] Beliard K, Ebekozien O, Demeterco-Berggren C, et al Increased DKA at presentation among newly diagnosed type 1 diabetes patients with or without COVID-19: data from a multi-site surveillance registry. J Diabetes. 2021;13(3):270‐272. 33283979 10.1111/1753-0407.13141

[12] Saydah SH, Shrestha SS, Zhang P, Zhou X, Imperatore G. Medical costs among youth younger than 20 years of age with and without diabetic ketoacidosis at the time of diabetes diagnosis. Diabetes Care. 2019;42(12):2256‐2261. 31575641 10.2337/dc19-1041 PMC10999225

[13] Rewers A, Dong F, Slover RH, Klingensmith GJ, Rewers M. Incidence of diabetic ketoacidosis at diagnosis of type 1 diabetes in Colorado youth, 1998-2012. JAMA. 2015;313(15):1570‐1572. 25898057 10.1001/jama.2015.1414

[14] Duca LM, Wang B, Rewers M, Rewers A. Diabetic ketoacidosis at diagnosis of type 1 diabetes predicts poor long-term glycemic control. Diabetes Care. 2017;40(9):1249‐1255. 28667128 10.2337/dc17-0558

[15] Fredheim S, Johannesen J, Johansen A, et al Diabetic ketoacidosis at the onset of type 1 diabetes is associated with future HbA1c levels. Diabetologia. 2013;56(5):995‐1003. 23389397 10.1007/s00125-013-2850-z

[16] Shalitin S, Fisher S, Yackbovitch-Gavan M, et al Ketoacidosis at onset of type 1 diabetes is a predictor of long-term glycemic control. Pediatr Diabetes. 2018;19(2):320‐328. 28568379 10.1111/pedi.12546

[17] Alonso GT, Ebekozien O, Gallagher MP, et al Diabetic ketoacidosis drives COVID-19 related hospitalizations in children with type 1 diabetes. J Diabetes. 2021;13(8):681‐687. 33855813 10.1111/1753-0407.13184 PMC8251108

[18] Insel RA, Dunne JL, Atkinson MA, et al Staging presymptomatic type 1 diabetes: a scientific statement of JDRF, the endocrine society, and the American diabetes association. Diabetes Care. 2015;38(10):1964‐1974. 26404926 10.2337/dc15-1419 PMC5321245

[19] APCD Council . All-Payer Claims Database Council. Accessed March, 2022, https://www.apcdcouncil.org

[20] Wolfsdorf JI, Glaser N, Agus M, et al ISPAD clinical practice consensus guidelines 2018: diabetic ketoacidosis and the hyperglycemic hyperosmolar state. Pediatr Diabetes. 2018;19:155‐177. 29900641 10.1111/pedi.12701

[21] Center for Improving Value in Health Care . CO APCD Overview. Accessed June 2, 2025, https://civhc.org/get-data/co-apcd-info/#:∼:text=The%20CO%20APCD%20is%20the%20state%E2%80%99s%20most%20comprehensive,Advantage%29%2C%20and%20Health%20First%20Colorado%20%28Colorado%E2%80%99s%20Medicaid%20program%29

[22] Zhong VW, Pfaff ER, Beavers DP, et al Use of administrative and electronic health record data for development of automated algorithms for childhood diabetes case ascertainment and type classification: the SEARCH for diabetes in youth study. Pediatr Diabetes. 2014;15(8):573‐584. 24913103 10.1111/pedi.12152 PMC4229415

[23] Flanagan BE, Gregory EW, Hallisey EJ, Heitgerd JL, Lewis B. A social vulnerability index for disaster management. J Homel Secur Emerg Manag. 2011;8(1):16‐17.

[24] Shen Y, Sommers BD, Hatfield LA, Hayes C, Pandya A, Menzies NA. Insurance dynamics during childhood in the fragmented US health system. JAMA. 2025;334:1533‐1540 . 40991296 10.1001/jama.2025.15488 PMC12461604

[25] Atolagbe OO, Romano PS, Southern DA, Wongtanasarasin W, Ghali WA. Coding rules for uncertain and “ruled out”. diagnoses in ICD-10 and ICD-11. BMC Med Inform Decis Mak. 2024;21(Suppl 6):386. 39334213 10.1186/s12911-024-02661-6 PMC11430383

